# Gonorrhœal Ulcero-Membranous Stomatitis

**Published:** 1905-04

**Authors:** Eugene S. Talbot

**Affiliations:** Chicago


### GONORRHŒAL ULCERO-MEMBRANOUS STOMATITIS.

BY EUGENE S. TALBOT, M.S., D.D.S., M.D., LL.D., CHICAGO.

SINCE Neisser discovered the gonococcus, in 1878, much light has been thrown upon obscure infection in different parts of the body. This germ is now known not to be confined to the urethral gonorrhœal lesions and discharges, but that nearly every structure, particularly mucous membrane, may become involved. Successful culture methods have added much to diagnosis.

The literature of gonorrhœal infection of the mouth in adults is meagre. W. L. Baum [1] remarks, "Gonorrhœal ulcero-membranous stomatitis is, according to Menard, always due to profound systemic infection, is always a secondary state. This position was opposed by J. P. Tuttle [2] more than a decade ago, who cited in opposition, among other cases, that of C. W. Cutler,[3] where, in an adult, osculation of a gonorrhœic penis was followed in a few hours by a raw, dry feeling in the mouth and in twenty-four hours by the lip vesicles. The gums by the third day were painfully swollen, and on the fifth the mouth was intensely inflamed. A whitish fluid having a disagreeable odor and taste was secreted. The lips cracked and were covered with herpes; the lip and cheek

---

[1] The Practical Medicine Series, vol. x., 1904.
[2] Morrow's System, vol. i., 1893.
[3] N. Y. Med. Jour., January 25, 1889.

mucous membrane was thickened, reddened, denuded of epithelium in spots, and in patches covered with a false membrane. Cases later reported by R. Larson [1] were of similar type and origin.

"Gonorrhœa, a common epidemic in institutions for children, usually assumes the form of ophthalmia, vulvo-vaginitis, and pyæmia. In a number of cases of infant pyæmia reported by R. B. Kimball,[2] no local lesion was found to explain the entrance of the gonococcus to the general circulation. In Kimball's opinion, the gonococcus may produce stomatitis, from which a systemic infection could arise. Many conditions of the gums and alveolar process, such as occur prior to pyorrhœa alveolaris, predispose to stomatitis, readily forming a culture medium for the gonococcus. Conditions like this in an infant can easily be mistaken for sprue and treated therefor, thus concealing a possible source of systemic infection resulting in pyæmia.

"Gonorrhœal stomatitis, in a case described by Juergens, developed on the gums and cheeks of a man who had recently had gonorrhœa. The resultant dirty gray deposit contained the gonococcus."

Two cases have come to my notice in the past six months. A thirty-six-year-old travelling salesman, unmarried, was sent to me by his physician for local treatment, May 3, 1904. There was a history of gonorrhœa with continual urethral discharge. The mucous membrane on the entire superior alveolar process was involved. The gingival border of the mucous membrane was destroyed and the alveolar process was exposed. Marked inflammation with excessive swelling occurred. Interstitial gingivitis extended throughout the alveolar process, and the teeth were loose. The pain was so severe that sleep was out of the question. Salivation was excessive. The raw ulceration was covered with a glazed surface of greenish-gray color. Local application of glycerol was used for three days, when he left the city, at which time he said the pain was not so severe. The time was too short to notice any improvement of the mucous membrane.

A forty-five-year-old clerk, married, came for consultation, who had contracted gonorrhœa twenty-four years before. He had suffered with gleet ever since. The anterior superior alveolar process

---

[1] St. Louis Med. and Surg. Jour., 1896.

[2] Med. Record, November 14, 1903.

was exceedingly inflamed and raw. It was badly swollen. He suffered severe pain. Interstitial gingivitis was very profuse through the alveolar process. The incisors were loose, two of which I was obliged to remove. The surface was ulcerated and raw with a glazed surface of greenish-gray color. He has been married eight years; has no children. His wife has had local trouble, for which she has had three operations; he does not know the nature of her trouble. He was advised by a former consultant to have his jaw removed, because of its cancerous appearance. Hektoen's examination of the tissue for cancer proved it to be simply inflammatory. I suspected gonorrhœal infection. Examination of the tissue proved my suspicion correct. Applications were first made of glycerol, then silver nitrate. The inflammation subsided in the anterior part of the mouth, but extended rapidy through the vault of the soft palate, which was reached in three weeks from the time I first saw him. On consultation with Dr. Baum, argyrol (twenty-five per cent.) is now used.

Both these men were in the best of health except slight rheumatism. Both seem to have been infected by the fingers as an intermediary host of the gonococcus.

A horny gonorrhœal exanthem is described by G. Baermann.[1] The exanthem was secondary to a severe gonorrhœal attack, and was hyperkeratotic in nature. It bore some resemblance to certain tertiary luetic results and to psoriasis. A bacillus was found which resembled that of the bacillus of the kerosis group. Baermann is inclined to refer the results to this bacillus, as the gonococcus was not found. It would seem more probable, Baum remarks, from the results of Woodruff,[2] that the gonotoxin, which is exceedingly irritating, was the proximate determining cause. The exanthem involved the upper surface of the foot as well as the sole, and was evidently related to the arthritic conditions which appeared in the case.

That the gonotoxin could be drawn to the alveolar process when the elimination is interfered with elsewhere is undeniable. That processes could be set up similar to the hyperkeratotic states ascribed by Baum to the gonotoxin is equally indisputable, still the evidence in my two cases pointed to direct infection.

---

[1] Arch. f. Derm. u. Syph., B. lxix.

[2] Practical Medicine Series, vol. x., 1902.

